# Acute lymphoblastic leukemia with bone marrow necrosis as the first clinical manifestation: a pediatric case report

**DOI:** 10.3389/fonc.2026.1737632

**Published:** 2026-01-22

**Authors:** Liangwu Pan, Jianren Lin, Xiaobo Zhou, Chuanming Huang, Yanghui Zeng, Ying Fu

**Affiliations:** Department of Pediatric Hematological Oncology, Shunde Women and Children’s Hospital of Guangdong Medical University, Foshan, China

**Keywords:** acute lymphoblastic leukemia, blinatumomab, bone marrow necrosis, case report, pediatric

## Abstract

This paper reports a rare case of a 4-year-old male child with acute lymphoblastic leukemia (ALL) presenting initially with bone marrow necrosis (BMN) as the chief clinical manifestation. The child sought medical attention due to fever, bone pain, and fatigue. Laboratory tests indicated pancytopenia. Initial bone marrow cytomorphology examination revealed disrupted cellular architecture, suggesting possible BMN, and single-site flow cytometry detected no definitive abnormalities, highlighting the diagnostic complexity caused by BMN. Through multi-site bone marrow aspiration and biopsy, the diagnosis was ultimately confirmed as common B-cell ALL (common-B-ALL). Treatment followed the South China Children’s Cancer Collaborative Group SCCCG-ALL-2023 protocol, incorporating blinatumomab immunotherapy based on risk stratification. The child responded well to treatment and is currently in the maintenance chemotherapy phase, with minimal residual disease (MRD) monitoring consistently indicating complete remission. This case emphasizes the importance of early recognition of rare presentations like BMN-onset in pediatric ALL, the necessity of multi-site bone marrow examination, and the crucial role of individualized treatment strategies.

## 1 Introduction

Acute lymphoblastic leukemia (ALL) is the most common malignant tumor in childhood, with an incidence rate of approximately 3-4/100,000 in children under 10 years old, and a slightly higher incidence in males than females (1). Typical clinical manifestations include fever, pallor, fatigue, and bleeding tendencies, often accompanied by infiltration of organs such as the liver, spleen, lymph nodes, and mediastinum. Bone marrow necrosis (BMN) is a rare clinical-pathological condition, and its presentation as the initial manifestation of ALL is particularly uncommon clinically. Literature reports indicate that the incidence of BMN in patients with malignant tumors is approximately 0.3%--2.2% (2). Its pathogenesis involves multiple factors such as leukemic cell infiltration of the bone marrow microenvironment, local ischemia, release of inflammatory factors, and chemotherapy toxicity (3). Since the early symptoms of BMN are non-specific, often manifesting as bone pain, fever, and cytopenia, it is prone to misdiagnosis or missed diagnosis, posing significant diagnostic challenges. Bone marrow aspiration and biopsy are key methods for diagnosing BMN. However, in cases of extensive necrosis, single-site aspiration often fails to obtain adequate specimens, necessitating multi-site aspiration and histological examination to improve diagnostic accuracy (4). This paper reports a case of a 4-year-old child with ALL initially presenting as BMN, where initial single-site bone marrow morphology and flow cytometry showed no clear evidence of malignancy, but common-B-ALL was ultimately diagnosed through multi-site bone marrow aspiration and biopsy. This case aims to enhance clinicians’ ability to recognize pediatric ALL presenting with BMN and provide references for optimizing related diagnosis and treatment strategies.

## 2 Case description

The patient is a 4-year-old male, preschool-aged, from Shunde, Guangdong, who presented at an external hospital outpatient clinic on October 27, 2023, due to “fever, bone pain, fatigue.” Because his blood tests showed abnormalities, his family brought him to our hospital’s pediatric hematology specialty for further evaluation on November 1, 2023. The patient’s past medical history, personal history, and family history were unremarkable.

Physical examination upon admission: Temperature 37 °C, heart rate 133 beats/minute, skin and mucosa appeared pale. A red hemangioma, approximately 4.0 × 4.0 cm in size, was visible on the left upper arm. The left ankle joint was tender, without local redness or swelling. Cardiovascular, respiratory, abdominal, and neurological examinations revealed no significant abnormalities.

Initial laboratory tests upon admission showed: White blood cell count 2.82 × 10^9^/L, neutrophils 0.55 × 10^9^/L, lymphocytes 2.41 × 10^9^/L, hemoglobin 90 g/L, platelets 98 × 10^9^/L. C-reactive protein was significantly elevated at 123.1 mg/L. Urinalysis, stool routine, Mycoplasma pneumoniae IgG and IgM antibodies, respiratory pathogen panel, T-spot, EBV DNA quantification, electrolytes, liver and kidney function, myocardial enzyme profile, erythrocyte sedimentation rate, and immunology-related tests were all within normal ranges. Electrocardiogram indicated sinus tachycardia. Abdominal ultrasound suggested hepatomegaly; gallbladder, spleen, pancreas, kidneys, and bladder showed no definite space-occupying lesions; heart structure was normal, left ventricular systolic function was normal. Chest CT indicated mild infectious signs in the lateral segment of the right middle lobe and the posterior basal segment of the left lower lobe. For a chronological overview of key clinical events and diagnostic procedures, see **Table 1**.

**Table 1:** Clinical timeline.

Timeline	Events
2023-10-27	Initial visit (external hospital) with chief complaints of fever, bone pain, and fatigue.
2023-11-01	Transferred to our hospital. Laboratory tests revealed pancytopenia.
2023-11-03	First bone marrow examination (left anterior superior iliac spine): Morphology showed extensive necrosis (BMN) with disrupted cellular architecture, making lineage identification difficult. Peripheral blood smear showed 1% blasts. Flow cytometry: No immunophenotypic abnormalities suggestive of acute leukemia, NHL, or high-risk MDS were detected. Preliminary diagnosis: Bone marrow necrosis (BMN).
2023-11-10	Multi-site bone marrow aspiration and biopsy: Final diagnosis confirmed as Common B-cell acute lymphoblastic leukemia (Common B-ALL).
2023-11-13	Initiated multi-phase intensive chemotherapy (VDLD induction chemotherapy) according to the SCCCG-ALL-2023 protocol (intermediate-risk group).
Day 15 Post-Chemotherapy (approx. 2023-11-27)	Bone marrow morphology and MRD assessment confirmed remission.
Day 33 Post-Chemotherapy (2023-12-15)	Bone marrow morphology and MRD assessment confirmed remission.
Post-Intensification Week 12 (2024-03-05)	Bone marrow morphology and MRD assessment at the end of intensification therapy (12 weeks) confirmed remission. Proceeded to consolidation chemotherapy. Pre-maintenance blinatumomab immunotherapy was administered based on risk stratification (5 µg/kg/day for 3 days → 15 µg/kg/day for 11 day). Tolerability: Good. No reported severe exacerbation of bone pain or BMN recurrence as described in some literature. Biochemical markers (ALP, LDH) showed no significant abnormalities.
2024-08-19	Initiated maintenance chemotherapy.
Subsequent Time Points	Currently in the maintenance therapy phase. Bone marrow morphology and MRD assessments are performed every 3 months, confirming ongoing complete remission.

**Figure 1:**
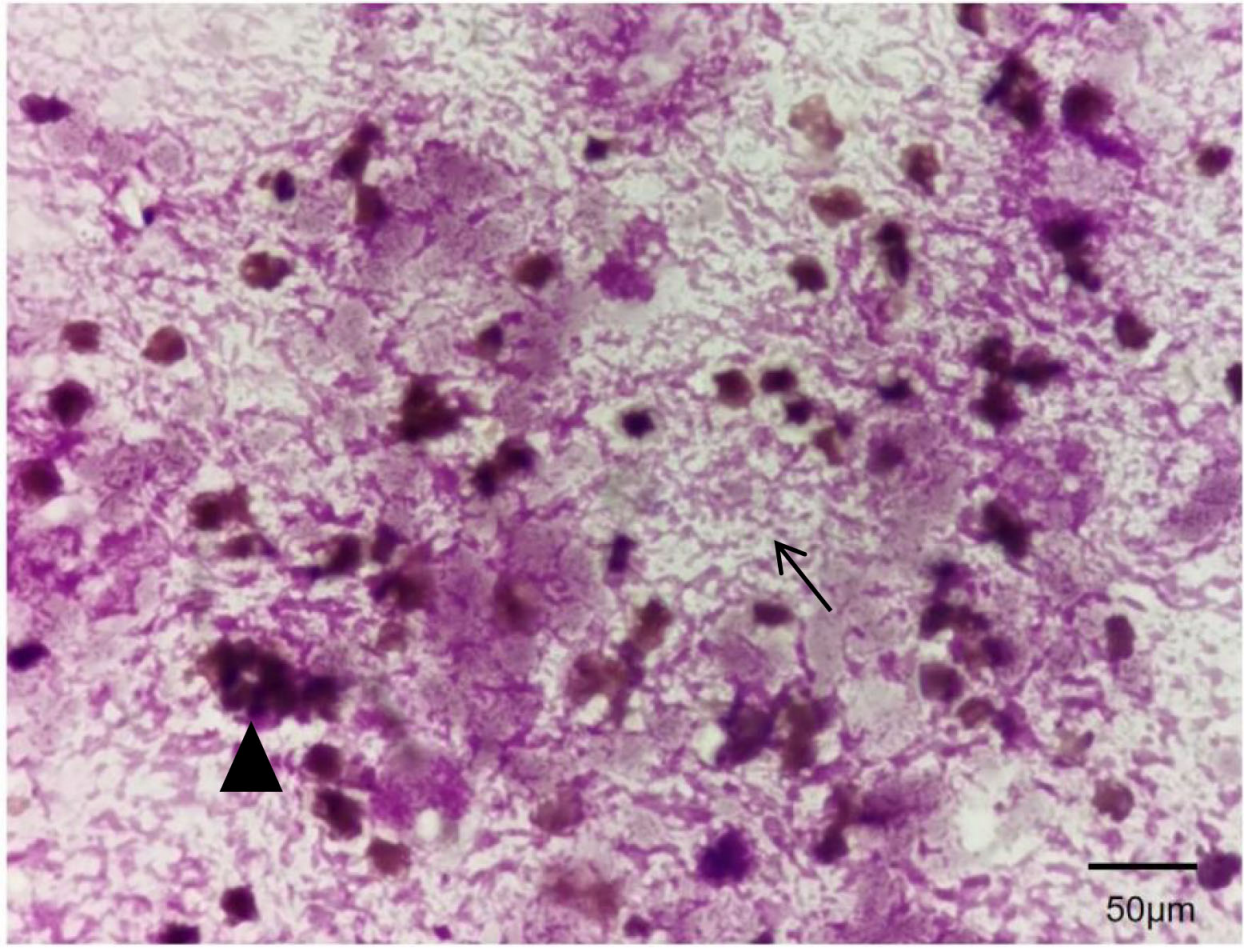
Bone marrow biopsy from the left iliac crest. The bone marrow demonstrates extensive cytomorphological abnormalities, with most nucleated cells exhibiting poorly defined contours, irregular cell borders, and indistinct nuclear-to-cytoplasmic differentiation (→), making lineage identification difficult. Numerous fragmented erythrocytes are present (▴), suggestive of bone marrow necrosis. Wright’s Stain, ×1000.

**Figure 2:**
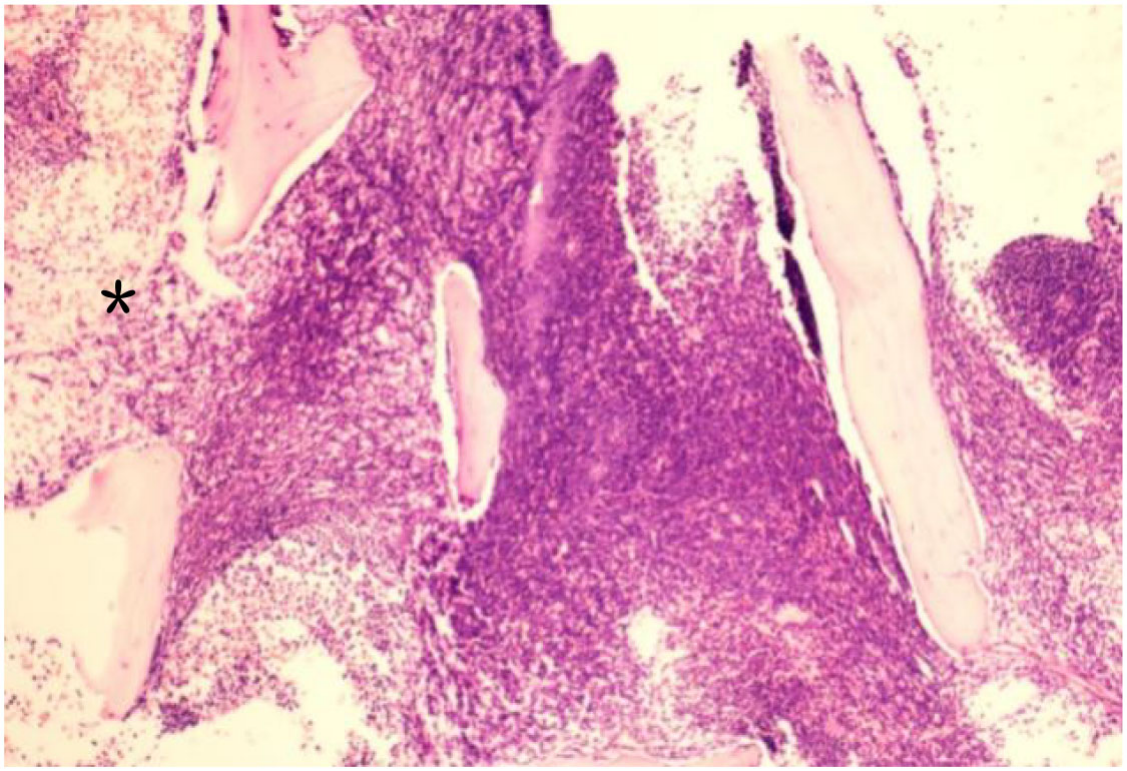
Bone marrow biopsy showing extensive necrosis (*) and disrupted hematopoietic architecture in the right field. H&E ×100.

Bone marrow examination performed on November 3, 2023: Bone marrow morphology from the left anterior superior iliac spine aspiration specimen showed that most nucleated cells had unclear contours, irregular borders, and indistinct nuclear-cytoplasmic structure, making lineage identification difficult, and fragmented mature red blood cells were visible, highly suggestive of bone marrow necrosis (BMN) (**Figure 1**, **Figure 2**). Peripheral blood smear morphology examination revealed 1% blasts. However, concurrent flow cytometry detected no evidence of immunophenotypic abnormalities associated with acute leukemia, non-Hodgkin lymphoma (NHL), or high-risk myelodysplastic syndrome (MDS).

## 3 Diagnosis and treatment

Based on the child’s clinical presentation and initial examination results, the preliminary diagnosis was bone marrow necrosis (BMN), bronchopneumonia, and hemangioma. Given the unknown etiology of BMN and high suspicion of an underlying hematological malignancy, an urgent case discussion was held within the department. To clarify the etiology, multi-site bone marrow aspiration and bone marrow biopsy were promptly arranged for comprehensive evaluation including morphology, flow cytometry, and genetics. During this period, the child received symptomatic supportive treatment including cefoperazone/sulbactam for anti-infection, nebulization, and expectorant therapy. His clinical symptoms gradually improved, and follow-up inflammatory markers returned to normal.

Multi-site re-evaluation (sternum and bilateral posterior superior iliac spines) revealed hypercellular marrow with 93% blasts in viable areas, confirming acute leukemia. Although hemodilution compromised right iliac spine sampling, left-sided specimens yielded diagnostic material. Further bone marrow evaluation results showed that both sternum and right posterior superior iliac spine aspirations indicated extremely hypercellular marrow with a high proportion of blasts up to 93%, highly suggestive of acute leukemia. The right posterior superior iliac spine aspiration specimen was affected by hemodilution, compromising assessment. Flow cytometry detected a population of abnormal blasts, accounting for approximately 76% of nucleated cells, with an immunophenotype: positive for HLA-DR, CD10, CD19, CD22, CD34, CD38, CD58, CD123, cytoplasmic CD79a, and TdT, with significant suppression of myeloid proliferation, consistent with acute B-lymphoblastic leukemia (common-B-ALL) characteristics. Bone marrow biopsy from the right anterior superior iliac spine showed extreme hyperplasia of hematopoietic tissue (approximately 90%), reduced adipose tissue, decreased granulocytic, erythroid, and megakaryocytic lineages, and diffuse proliferation of medium-sized blasts, with BMN and focal fibrous tissue hyperplasia visible in some areas. Immunohistochemistry results: CD3 scattered positive, Pax-5, CD79a, and TdT diffusely positive, MPO scattered positive. The pathological conclusion was consistent with B-ALL, recommending comprehensive diagnosis combined with other tests. The final diagnosis was acute lymphoblastic leukemia. Cytogenetic examination revealed no abnormal karyotype, while molecular genetic testing using a reverse transcription-polymerase chain reaction (RT-PCR) assay detected the ETV6/RUNX1 gene fusion.

The patient officially began treatment on November 13, 2023, following the South China Children’s Cancer Collaborative Group SCCCG-ALL-2023 protocol (a multicenter prospective cohort study, registration number ChiCTR2300075242), using the VDLD regimen (Vincristine, Daunorubicin, L-Asparaginase, Dexamethasone) for induction chemotherapy. Bone marrow morphology and minimal residual disease (MRD) assessment on day 15 of chemotherapy confirmed achievement of hematological remission.

According to the SCCCG-ALL-2023 protocol risk stratification, this child was classified into the intermediate-risk group due to the presence of BMN at initial diagnosis (5). Subsequently, he received corresponding intensive chemotherapy, including additional induction, consolidation, and reinduction phases. To minimize the risk of relapse, a 14-day course of blinatumomab immunotherapy was combined with chemotherapy (6, 7). This bispecific T-cell engager targets CD19/CD3, enhancing T-cell mediated killing of leukemic B-cells to eliminate MR. The 14-day regimen (5 µg/kg/day for 3 days → 15 µg/kg/day for 11 day) was selected based on the SCCCG-ALL-2023 protocol for intermediate-risk patients, aiming to optimize efficacy while mitigating potential risks associated with bone marrow necrosis, such as exacerbation of bone pain or biochemical abnormalities reported in literature (6).During treatment, the child tolerated blinatumomab well, without severe bone pain, edema, or significant elevation of serum alkaline phosphatase (ALP) or lactate dehydrogenase (LDH) – abnormal biochemical changes associated with BMN reported in the literature (6). The child is currently in the maintenance chemotherapy phase, and regular MRD monitoring consistently indicates that the disease remains in complete remission (CR).

## 4 Discussion

Bone marrow necrosis (BMN) is a rare clinical-pathological syndrome first described by Wade and Stevenson in 1941 in patients with sickle cell anemia (8). Its pathological basis involves varying degrees of necrosis of the bone marrow hematopoietic tissue and stroma. Literature reports indicate that the incidence of BMN in patients with malignant tumors is approximately 0.3% to 2.2% (2). Common clinical manifestations include bone pain, fever, and cytopenia. In this case, the child presented with fever, bone pain, and peripheral pancytopenia. Initial bone marrow morphology suggested BMN, which is consistent with reports in the literature.

The diagnosis of BMN primarily relies on bone marrow aspiration and biopsy. However, extensive necrosis can lead to failed bone marrow aspiration or non-diagnostic results (4). In this case, the initial left anterior superior iliac spine aspiration was difficult to interpret due to disrupted cellular architecture, and flow cytometry detected no abnormal phenotype, highlighting the limitations of single-site examination. According to the views of scholars like Knupp et al., extensive BMN requires multi-site aspiration and biopsy to improve the diagnostic rate (4). Through multi-site aspiration and biopsy of the sternum and both iliac bones, we ultimately confirmed the diagnosis of common-B-ALL in the extremely hypercellular areas and observed focal necrosis and fibrosis, confirming the necessity of multi-site examination in the diagnosis of BMN-associated leukemia. According to the views of scholars like Knupp et al., extensive BMN requires multi-site aspiration and biopsy to improve diagnostic accuracy (4), as demonstrated in our case where multi-site examination proved critical for confirming common-B-ALL diagnosis despite initial inconclusive findings.

The etiology of BMN is diverse, including hematological malignancies, solid tumors, infections, sickle cell anemia, and other factors (3). In ALL, its pathogenesis is complex. Firstly, the excessive proliferation of leukemic cells within the bone marrow cavity depletes nutrients and oxygen, competes for living space, and directly disrupts the homeostasis of the bone marrow microenvironment, potentially leading to necrosis (9). Secondly, leukemia can activate the coagulation system, triggering disseminated intravascular coagulation (DIC), leading to microvascular embolism and local ischemic hypoxia in the bone marrow, thereby inducing necrosis (10). Furthermore, immune-inflammatory responses are also involved; the release of pro-inflammatory cytokines such as tumor necrosis factor-alpha (TNF-α) may exacerbate tissue damage (11, 12). In this case, no other secondary factors such as infection or coagulation abnormalities were found, therefore BMN was considered primarily caused by the leukemic infiltration of the bone marrow microenvironment itself.

The core of BMN treatment lies in actively controlling the primary disease. After diagnosis, this child immediately received chemotherapy according to the SCCCG-ALL-2023 protocol and, based on risk stratification (classified as intermediate risk due to concomitant BMN), received multi-phase intensive chemotherapy including VDLD induction, consolidation, reinduction, and maintenance. Notably, to enhance efficacy and eliminate minimal residual disease (MRD), blinatumomab was combined with the treatment. Blinatumomab is a bispecific T-cell engager that, by binding CD19 and CD3, directs T cells to specifically kill leukemic B-cells (7). Some literature reports that BMN or exacerbation of bone pain may occur during blinatumomab treatment (6). This case did not observe similar severe adverse reactions or abnormalities in biochemical markers (such as ALP, LDH) during blinatumomab treatment; tolerance was good, and sustained complete remission was ultimately achieved.

Studies suggest that leukemia patients with BMN may have a poorer prognosis, with survival rates affected (5). The presence of BMN often indicates high tumor burden and severe destruction of the bone marrow microenvironment, which may be associated with poor treatment response and increased risk of relapse. Therefore, for such patients, adopting a more aggressive, multimodal treatment strategy and closely monitoring MRD are crucial for improving outcomes. Notably, the ETV6/RUNX1 fusion gene detected in this case is typically associated with a favorable prognosis in pediatric ALL, characterized by high remission rates and lower relapse risk (13–17). However, in the context of concomitant BMN—which may indicate adverse factors like microenvironment disruption—the overall prognostic impact requires careful individualized assessment (18). This highlights the importance of integrating genetic markers with clinical presentations for risk stratification.

This case, as an instance of pediatric ALL initially presenting as BMN, holds significant clinical implications. Firstly, for children presenting with unexplained bone pain, fever, and cytopenia, clinicians should be vigilant about the possibility of BMN and regard it as an important signal of potential hematological malignancy. Secondly, when there is high clinical suspicion of malignancy but initial bone marrow aspiration is negative or suggests BMN, the diagnosis should not be easily ruled out. Instead, active pursuit of multi-site bone marrow aspiration and biopsy is key to avoiding misdiagnosis and missed diagnosis (4, 19, 20). Finally, for children diagnosed with ALL complicated by BMN, an intensive treatment plan should be formulated based on standardized risk stratification, and consideration can be given to combining targeted immunotherapy to maximize MRD clearance and improve long-term prognosis (21, 22).

## 5 Conclusions

This case report details a pediatric case of acute lymphoblastic leukemia (ALL) presenting initially with bone marrow necrosis (BMN) as the chief clinical manifestation. BMN is a rare and diagnostically challenging pathological condition, whose pathogenesis is related to multiple factors including leukemic cell infiltration causing bone marrow microenvironment disruption, local ischemia, and inflammatory responses (9–11). The diagnosis and treatment process of this child highlights the insidious nature and diagnostic complexity of BMN – initial single-site bone marrow aspiration was difficult to diagnose due to cellular structure disruption, and common-B-ALL with ETV6/RUNX1 fusion gene was ultimately confirmed through multi-site bone marrow aspiration and biopsy.

This case provides important insights for clinical practice: For children presenting with unexplained bone pain, fever, and cytopenia, the possibility of BMN should be suspected and regarded as a warning sign of potential hematological malignancy. At the diagnostic level, when clinical suspicion of leukemia is high but initial bone marrow findings are atypical or suggest necrosis, multi-site bone marrow aspiration and biopsy are key to improving diagnostic accuracy and avoiding delay (4, 19, 20). At the treatment level, the presence of BMN may indicate a relatively poor prognosis (5). Therefore, based on standardized risk stratification, an intensive comprehensive treatment plan including chemotherapy and targeted immunotherapy (such as blinatumomab) should be implemented, aiming to effectively clear minimal residual disease and improve patient survival outcomes (6, 7, 21, 22).

In the future, it is necessary to conduct further multicenter clinical studies to deeply explore the molecular mechanisms, risk prediction models, and optimized treatment strategies for BMN associated with pediatric ALL, thereby providing more substantial evidence support for the precise diagnosis and treatment of this special clinical presentation.

## Data Availability

The original contributions presented in the study are included in the article/supplementary material. Further inquiries can be directed to the corresponding author.
